# Greater Abdominal Fat Accumulation Is Associated with Higher Metabolic Risk in Chinese than in White People: An Ethnicity Study

**DOI:** 10.1371/journal.pone.0058688

**Published:** 2013-03-14

**Authors:** Wei He, Sha Zhang, Aihua Song, Min Yang, Jingjing Jiao, David B. Allison, Steven B. Heymsfield, Shankuan Zhu

**Affiliations:** 1 Obesity and Body Composition Research Center, Chronic Disease Research Institute, Zhejiang University School of Public Health, Hangzhou, China; 2 Department of Nutrition and Food Hygiene, Zhejiang University School of Public Health, Hangzhou, China; 3 Department of Biostatistics, and Nutrition Obesity Research Center, University of Alabama at Birmingham, Birmingham, Alabama, United States of America; 4 Pennington Biomedical Research Center, Baton Rouge, Louisiana, United States of America; Charité University Medicine Berlin, Germany

## Abstract

**Introduction:**

Chinese are reported to have a higher percent body fat (%BF) and a higher percent trunk fat (%TF) than whites for a given body mass index (BMI). However, the associations of these ethnic differences in body composition with metabolic risks remain unknown.

**Methods and Procedures:**

A total of 1 029 Chinese from Hangzhou, China, and 207 whites from New York, NY, USA, were recruited in the present study. Body composition was measured using dual-energy X-ray absorptiometry (DXA). Analysis of covariance was used to assess the ethnic differences in fat, fat distribution, and metabolic risk factors.

**Results:**

After adjusting for BMI, age, and height, Chinese men had an average of 3.9% more %BF and 12.1% more %TF than white men; Chinese women had an average of 2.3% more %BF and 11.8% more %TF than white women. Compared with whites, higher metabolic risks were detected in Chinese for a given BMI after adjusting for age and height. Further adjustment for %BF did not change these ethnic disparities. However, after adjusting for %TF, the ethnic differences decreased and become insignificant in triglyceride, high-density lipoprotein cholesterol, and blood pressure (except for systolic blood pressure in men). For fasting plasma glucose, the ethnic differences persisted after adjustment for %BF, but decreased significantly from 0.910 to 0.686 mmol/L among men, and from 0.629 to 0.355 mmol/L among women, when the analyses were further controlled for %TF.

**Discussion:**

Chinese have both higher %BF and %TF than white people for a given BMI. However, only %TF could in part account for the higher metabolic risk observed in Chinese men and women.

## Introduction

Obesity is a major worldwide public health problem owing to its close association with morbidity and mortality and its high prevalence in both developed and developing countries [1]–[3]. Body mass index (BMI, kg/m^2^) is a simple index of weight-for-height that is commonly used to classify underweight, overweight, and obesity in adults [1]. Lower cutoffs for overweight (BMI≥24 kg/m^2^) and obesity (BMI≥28 kg/m^2^) have been recommended for Chinese people by the China Obesity Task Force [4], [5]. However, this lower definition was challenged by a recent study in Taiwanese adults by Lin [6] and colleagues, who reported that the lowest risk of death was among adults with a BMI of 24.0 to 25.9 (mean, 24.9 kg/m^2^).

Despite the important findings by Lin and colleagues, a related commentary by our group suggested that caution should be exercised when considering raising the BMI cutoffs for Chinese people [7]. One basis for our argument was that Chinese persons are reported to have a higher percentage of body fat and more abdominal fat accumulation than do white persons for a given BMI [7]–[10]. However, whether these ethnic differences in body composition phenotypes will augment metabolic dysfunction in Chinese or whether they can explain the ethnic differences in the relationship between BMI and obesity-related risk factors remains unknown.

Higher body fat and more central fat accumulation have been hypothesized to be associated with the higher metabolic risk for a given BMI in Chinese people. Thus, using data collected on 1 029 Chinese adults at Zhejiang University, China, and 207 white adults at Columbia University, New York, NY, USA, this analysis aims to investigate the ethnic differences in the association of BMI with percent body fat (%BF) and percent trunk fat (%TF) and the relationship of these variables with metabolic risk factors.

## Materials and Methods

### Study Sample

**Figure 1:**
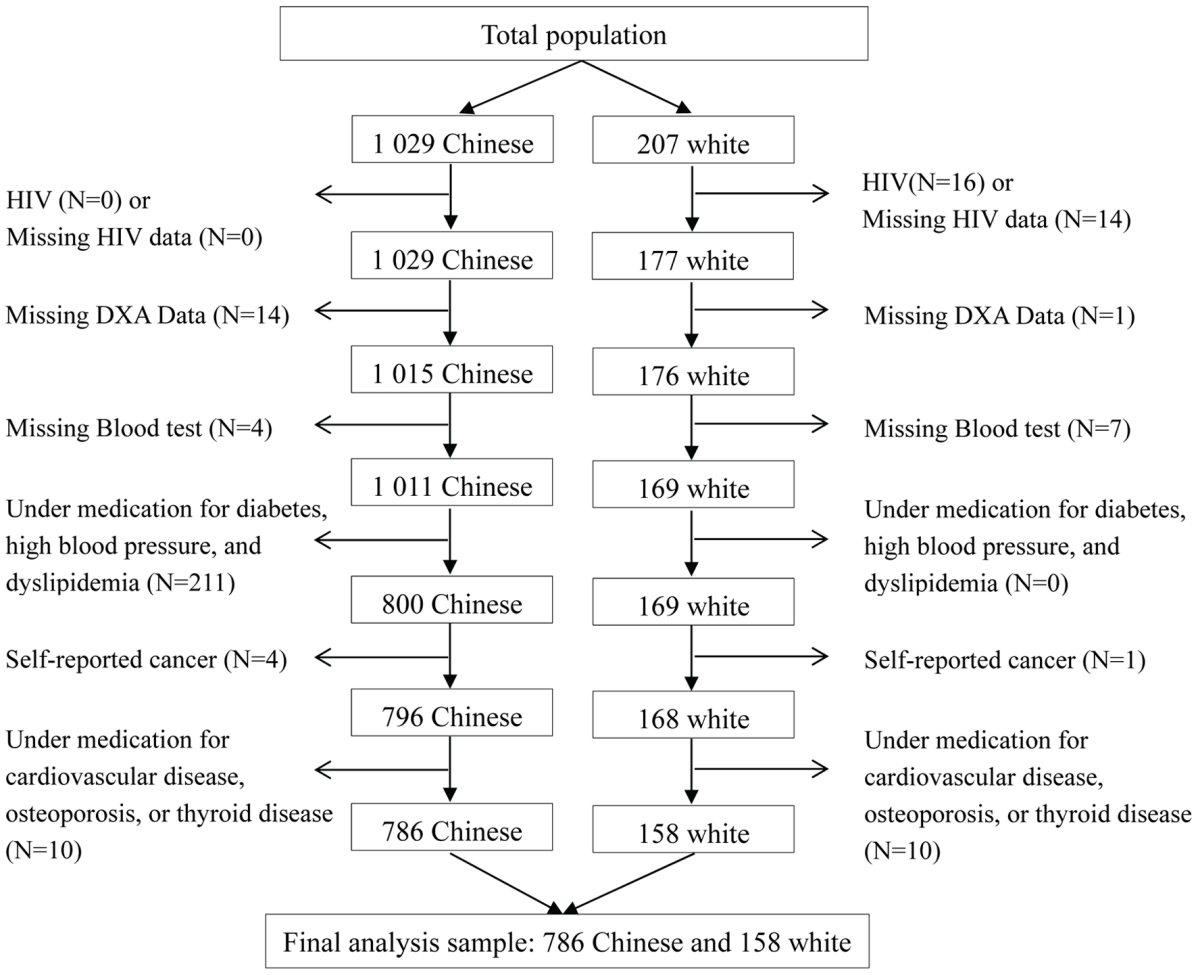
Flowchart of participant selection. DXA, dual-energy X-ray absorptiometry.

A total of 1029 Chinese and 207 white subjects were included in this study. Chinese participants were recruited and then voluntarily enrolled between November 2008 and May 2009 through the distribution of leaflets and posters provided by the local department of the Center for Disease Control and Prevention in Hangzhou, China. The data on whites were obtained between August 1998 and January 2001 from St. Luke’s-Roosevelt Hospital Center, Columbia University in New York, NY, USA. Details of these samples have been previously described [11]. In the present study, the following persons were excluded from the analysis: 30 subjects with positive HIV test or missing data on HIV; 15 subjects with missing dual-energy X-ray absorptiometry (DXA) values; 11 subjects with missing blood tests; 211 subjects on medication for hypertension, dyslipidemia, or diabetes; 5 subjects with self-reported cancer, and 20 subjects who were at the time on medication for cardiovascular diseases, osteoporosis, or thyroid disease. The final sample consisted of 387 men (321 Chinese and 66 white) and 557 women (465 Chinese and 92 white). The flow of participants included in the analysis is presented in Figure 1. In addition, blood pressure data from 66 white participants (25 men and 41 women) were unavailable; thus, only 41 white men and 51 white women were included in the blood pressure analysis. Signed informed consent was obtained from all subjects. The study was approved by the internal review boards of the Second Affiliated Hospital of Zhejiang University School of Medicine in Hangzhou, China, and St. Luke’s-Roosevelt Hospital Center in New York, NY, USA.

### Measurements

After a medical history questionnaire was administered and subjects had fasted overnight, venipuncture was conducted to permit measurement of fasting plasma glucose (FPG), triglyceride (TG), and high-density lipoprotein cholesterol (HDL-C). A certificated clinical laboratory at each site analyzed the blood samples. Weight was recorded to the nearest 0.1 kg and height was recorded to the nearest 0.1 cm. BMI was defined as weight in kilograms divided by the square of height in meters. Systolic blood pressure (SBP) and diastolic blood pressure (DBP) were measured by trained examiners with a mercury sphygmomanometer according to a standard protocol [12]. For each subject, two consecutive readings of blood pressure were taken in the right arm after five minutes of rest, with the participant in a seated position. The mean of the two measurements was used for analysis.

Total and trunk fat mass were measured by use of the same DXA system (Lunar Prodigy, Madison, WI, USA) at the Chinese and US sites as previously described [11]. The DXA machine was calibrated against a calibration phantom before each use, with a precision error of ≤0.8% for body fat in Zhejiang University and 3∼4% in the Obesity Research Center at Columbia University. To investigate any inter-machine variation, the body composition of one man and one woman was measured at each site within one month, with a coefficient of variation (CV) of 3.05% for total fat mass and a CV of 1.41% for trunk fat mass. %BF, an indication of general adiposity, was calculated as body fat divided by body weight. %TF was calculated as trunk fat divided by body fat and was used as a surrogate marker for central fat distribution.

### Statistical Analysis

All analyses were performed separately for males and females. Continuous data were presented as means with standard deviations. Differences between ethnic groups were tested by using Student’s t-test.

Analysis of covariance (ANCOVA) was used to assess the ethnic difference in %BF and %TF, unadjusted or adjusted for covariates. The associations between metabolic risk factors and ethnicity (with white race as the reference) were also examined by using ANCOVA, first with adjustment for age, BMI, and height and then with further adjustment for %BF or %TF. Tests for ethnic differences derived from these models were then performed by using the Wald test (as implemented in the Stata “test” module). To explore whether our subject selection procedures would affect our results, we also performed the analyses including subjects diagnosed with active disease (except for HIV) or on any medication. In addition, because some of the variables were severely skewed, natural log transformation, followed by Box-Cox transformation if necessary, was applied to the data to evaluate whether the skewed distributions would affect the results. Furthermore, a sensitivity analyses replacing BMI with body weight were also conducted. Logistic regression analyses were also applied to investigate the ethnic difference in odds ratio for having metabolic risk factors. Because of our small sample size, the outcome was defined as having at least one of the following components: *1)* raised TG lever (≥1.7 mmol/L), *2)* reduced HDL-cholesterol (<1.03 mmol/L in men and <1.29 mmol/L in women), *3)* raised fasting plasma glucose (≥5.6 mmol/L), or *4)* raised blood pressure (systolic blood pressure≥130 or diastolic blood pressure≥85 mm Hg). The level of significance for all statistical tests was set at 0.05 (two-tailed) unless otherwise indicated. All analyses were performed by using Stata version 11.0 (Stata Corporation, College Station, TX, USA).

## Results

No significant differences in age were detected between Chinese and white participants (**Table 1**). The whites were heavier, taller, and had higher BMIs than the Chinese. Despite the lower levels of body fat, trunk fat, and %BF in Chinese men and women, %TF was higher in Chinese than in whites (*p*<0.001 for both). For metabolic risk factors, Chinese men had significantly higher FPG (*p*<0.001), SBP (*p*<0.001), TG (*p* = 0.005), and HDL-C (*p* = 0.021), and insignificantly higher DBP (*p* = 0.104) than white men. Similar higher metabolic risks were also detected in Chinese women. In addition, Chinese women had lower HDL-C than white women (*p* = 0.005).

**Table 1:** Characteristics of the subjects by gender and ethnicity^1^.

	Men (*n* = 387)		Women (*n* = 557)	
	White	Chinese	White	Chinese
Sample size (n)	66	321	92	465
Age (y)	44.4(15.6)	47.9(14.3)	45.5(17.0)	45.9(13.0)
Anthropometric measurement				
Weight (kg)	84.7(12.3)	65.6***(10.2)	67.9(15.3)	56.5***(8.3)
Height (cm)	178.2(7.1)	167.5***(6.0)	163.5(7.3)	156.7***(5.6)
BMI (kg/m^2^)	26.7(3.8)	23.4***(3.1)	25.4(5.6)	23.0***(3.1)
Body fat mass (kg)	19.5(9.0)	13.6^***^(6.7)	23.6(11.9)	17.9***(5.6)
Trunk fat mass (kg)	10.5(5.3)	8.6**(4.4)	10.7(5.8)	9.9(3.5)
%BF	22.3(8.2)	19.9*(7.4)	32.(10.3)	31.0*(5.9)
%TF	52.8 (6.8)	61.4*** (5.9)	44.5 (5.5)	54.9*** (5.2)
Metabolic risk factors^2^				
FPG (mmol/L)	4.92(0.74)	5.55***(1.01)	4.77(0.57)	5.39***(0.84)
SBP(mmHg)	118(10)	126***(16)	110(19)	119***(17)
DBP (mmHg)	77(9)	79(10)	70(8.3)	75***(9)
TG (mmol/L)	1.18(0.70)	1.68**(2.74)	0.97(0.60)	1.23***(0.87)
HDL-C (mmol/L)	1.18(0.29)	1.27*(0.28)	1.54(0.42)	1.41**(0.32)

1 All continuous values are presented as means with standard deviations. %BF, body fat divided by body weight; %TF, trunk fat divided by body fat; FPG, fasting plasma glucose; SBP, systolic blood pressure; DBP, diastolic blood pressure; TG, triglyceride; HDL-C, high-density lipoprotein cholesterol. Differences between ethnic groups were tested by using Student’s t-test. *P* values for the difference between Chinese and white subjects: *<0.05, **<0.01, ***<0.001.

2 A total of 158 whites (66 men and 92 women) were included for the analysis of TG, HDL-C, and FPG, and 92 whites (41 men and 51 women) were included for the analysis of BP.

**Figure 2:**
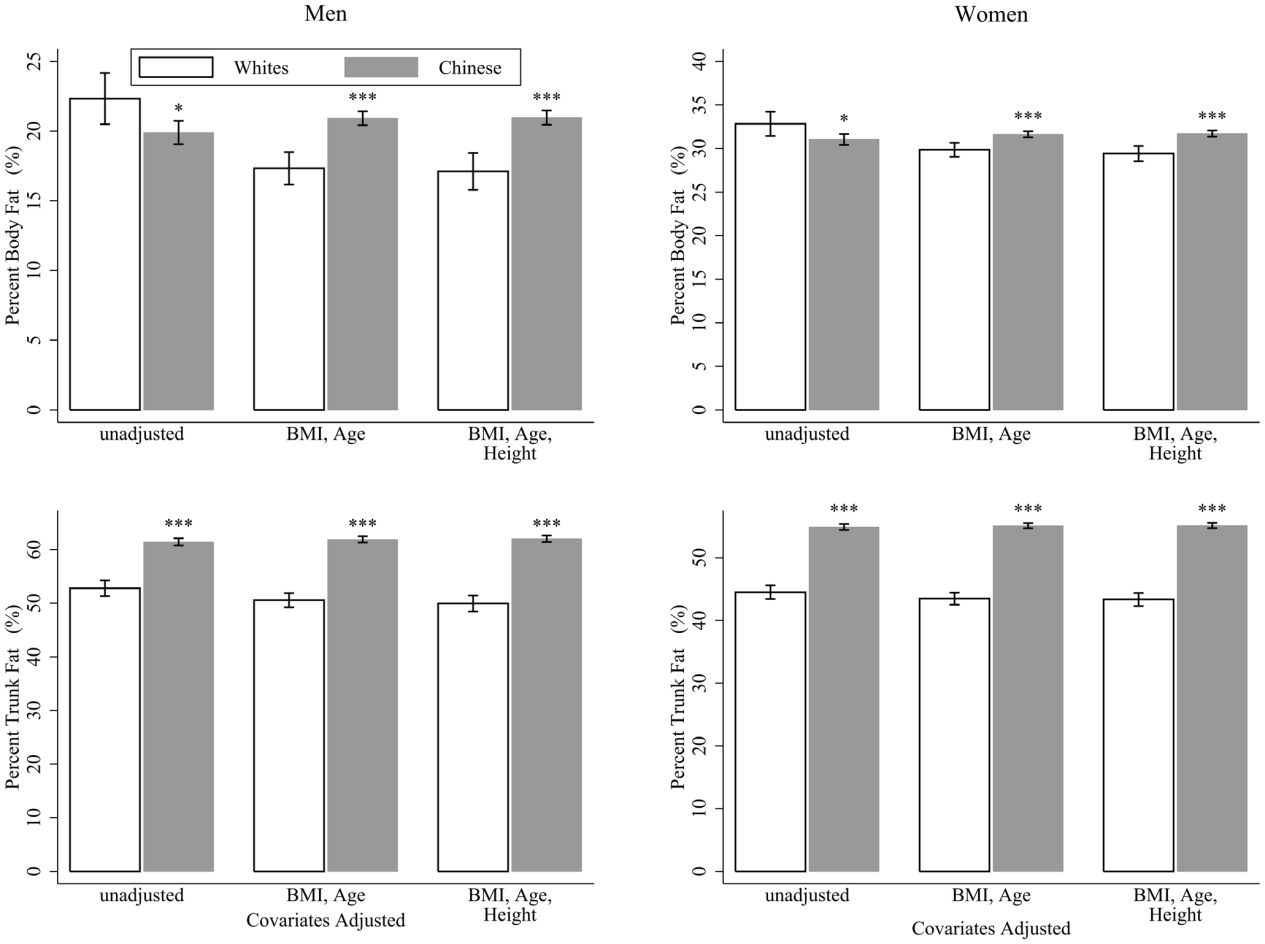
Percentage body fat and percentage trunk fat by ethnicity in men and women. Error bars represent 95% confidence intervals. Data were analyzed by using analysis of covariance (ANCOVA) with “ethnicity” as the grouping variable and the listed variables as covariates. BMI, body mass index. *p<0.05, **p<0.01, ***p<0.001, for the difference with white men or women, respectively.

The mean %BF and mean %TF by ethnicity derived from the ANCOVA analysis are presented in **Figure 2**. Compared with white adults, Chinese had lower %BF but higher %TF before adjustment. However, after correction for age, BMI, and height, Chinese had both higher %BF and higher %TF than did the whites, with an average of 3.9% and 12.1% more in men, and 2.3% and 11.8% more in women, respectively. Consistent results were found when we further included subjects with disease (except for HIV) or on medication (Figure S1).

**Figure 3:**
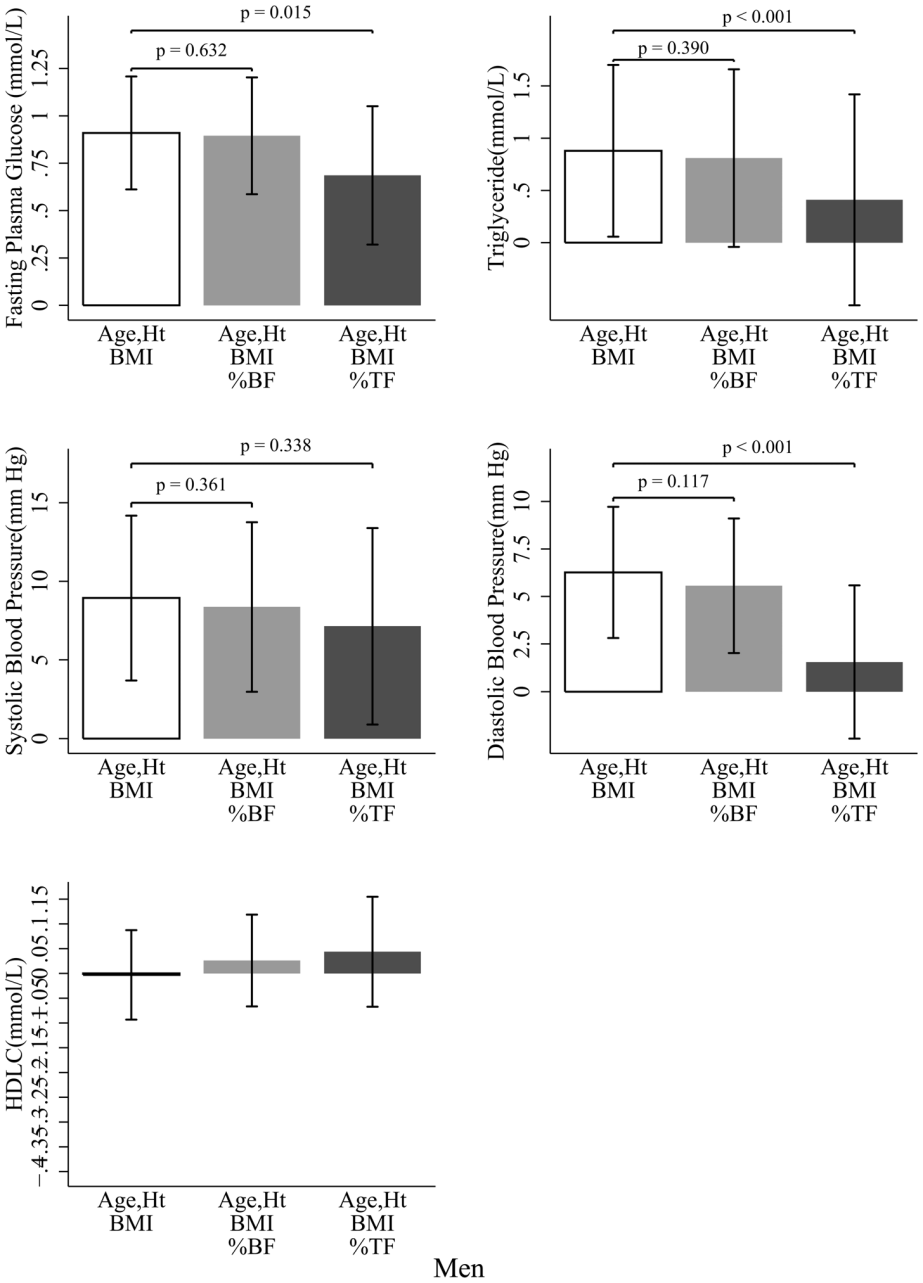
Ethnic differences (Chinese - white) in metabolic risk factors in men. Bar heights indicate the mean difference, whereas error bars represent 95% confidence intervals. Data were analyzed by analysis of covariance (ANCOVA) with “ethnicity” as the grouping variable and the listed variables as covariates. Ht, height; BMI, body mass index; %BF, body fat divided by body weight; %TF, trunk fat divided by body fat.

**Figure 4:**
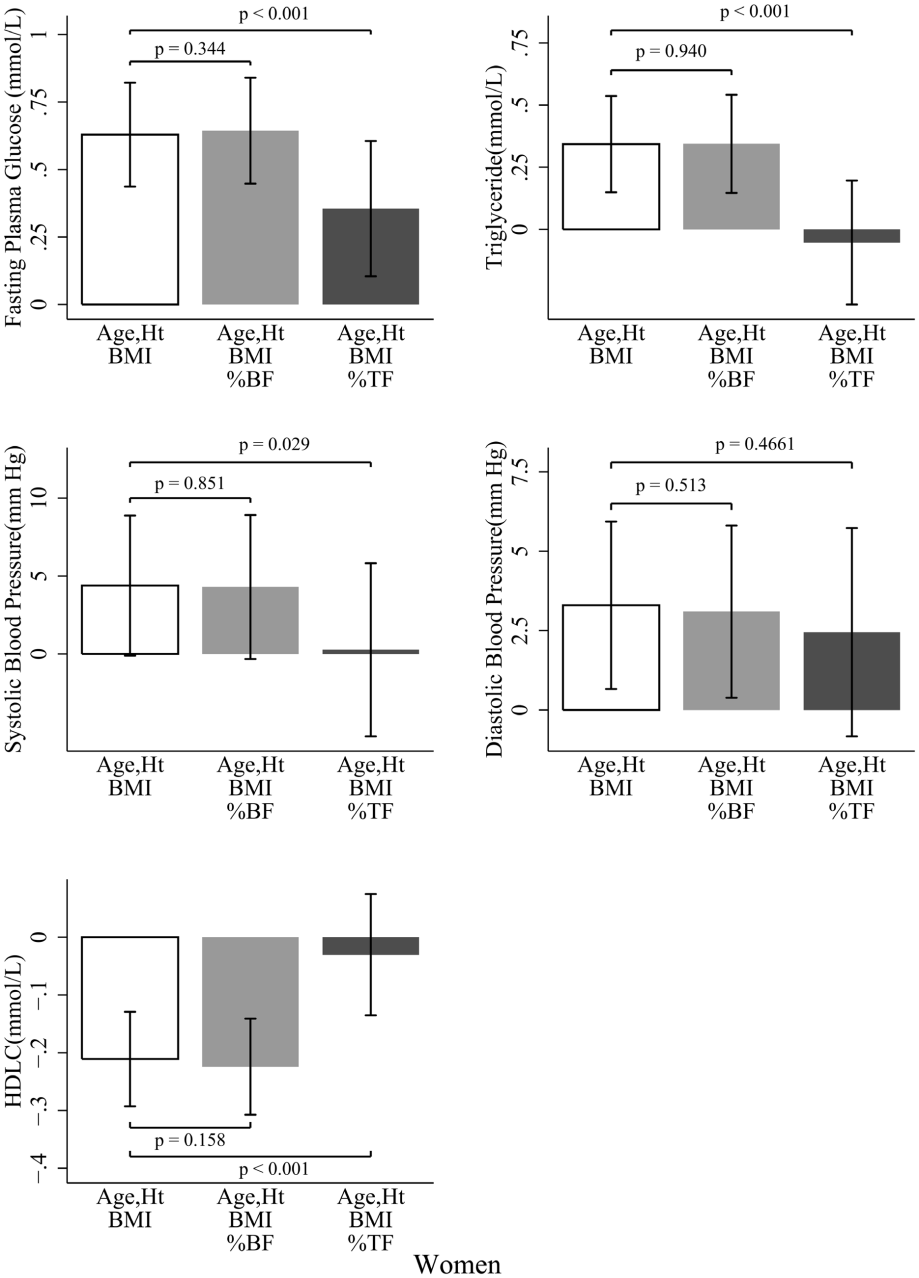
Ethnic differences (Chinese - white) in metabolic risk factors in women. Bar heights indicate the mean difference, whereas error bars represent 95% confidence intervals. Data were analyzed by analysis of covariance (ANCOVA) with “ethnicity” as the grouping variable and the listed variables as covariates. Ht, height; BMI, body mass index; %BF, body fat divided by body weight; %TF, trunk fat divided by body fat.

The metabolic risk factor differences between Chinese and whites before and after adjusting for %BF or %TF in men are presented in **Figure 3**. Chinese men had higher FPG (*p*<0.001), TG (*p* = 0.036), SBP (*p*<0.001), and DBP (*p*<0.001) than did white participants for a given BMI after control for age and height. Further adjustment for %BF did not attenuate these ethnic disparities, with no significant difference observed before or after the %BF adjustment were made. However, after adjustment for %TF, the ethnic differences in TG and DBP became insignificant (*p* = 0.425 for TG and *p* = 0.448 for DBP), along with a significant reduction in FPG (*p* = 0.015), TG (*p*<0.001), and DBP (*p*<0.001) except for SBP (*p* = 0.338). No significant ethnic differences in HDL-C were observed in any of these models in men. Similar results were detected in women (**Figure 4**). In addition, HDL-C did not change significantly in the %BF adjustment model. However, in the %TF adjustment model, the ethnic difference (Chinese – white) in HDL-C decreased significantly from −0.211 (*p*<0.001) to −0.030 (*p* = 0.571) mmol/L in women. In both men and women, %TF was significant in most of the models, whereas %BF was not. Including subjects diagnosed with active disease or on any medication did not change our findings (Figure S2, Figure S3). After logarithmic or Box-Cox transformation of the skewed variables, or replacing BMI with body weight, our results did not appreciably change (data not shown). Higher risk for having at least one of the metabolic risk factors were also observed in Chinese men (odds ratio, 5.12; *P*<0.001) and Chinese women (odds ratio, 2.60; *P* = 0.002) after adjusting for BMI, age, and height. This odds ratio decreased to 3.69 in the BF% adjustment model and to 1.48 in the %TF adjustment model in men, and to 2.98 for %BF model and to 0.64 for %TF model in women, respectively.

## Discussion

In this study, %BF and %TF were found to be higher in Chinese participants compared with whites, indicating that the Chinese accumulated more fat, especially in the region of the trunk, for a given BMI. Higher metabolic risks were also detected in the Chinese. However, most of these ethnic disparities in metabolic dysfunction could be partially accounted for by further controlling for %TF, an indication of central fat distribution.

Over the past decades, the prevalence of overweight and obesity, although still relatively lower than in Western countries such as the United States, has increased substantially in China [7]. Between 1992 and 2002, the prevalence of overweight in China increased by 38.6%, and the prevalence of obesity by 80.6%, which corresponds to another 70 million overweight and 30 million obese persons [13]. The development of overweight and obesity in China will likely increase the prevalence of some chronic diseases in the near future [14]. Multiple and integrated interventions are needed to reverse these trends in China.

To facilitate evaluation, an appropriate definition of obesity is needed for Chinese adults [15]. The BMI cutoffs recommended by the World Health Organization (WHO) to define overweight (≥25 kg/m^2^) and obesity (≥30 kg/m^2^) are primarily based on data from white populations [16], and thus may not be applicable for other ethnic groups [1], [17]. For Chinese people, lower cutoffs for overweight (≥24 kg/m^2^) and obesity (≥28 kg/m^2^) have been recommended by the China Obesity Task Force [4]. However, these criteria have been challenged by several recent studies that support the use of universal BMI cutoffs among different racial and ethnic groups [6], [18]. The present results suggest that ethnic-specific BMI cutoffs might be appropriate, because compared with the white participant, Chinese people have been reported to have more total and trunk fat mass, and a higher risk for obesity-related disorders, for a given BMI.

Comparing body fat and body fat distribution among ethnicities may be essential for the determination of the appropriateness of ethnic-specific BMI cutoffs because obesity is defined as an excess of fat mass [1], not as an excess of body weight, and abdominal adiposity is more strongly associated with risk factors for cardiovascular disease than is adipose tissue in other regions [19]. Using data from 115 Chinese and 114 white male pilots living in China, a recent study found that the Chinese men had more body fat and a greater degree of central fat deposition than the white men [10], which is consistent with the results found here and with those of many previous studies [9], [20], [21].

In addition to body composition, morbidities should also be considered for the determination of appropriate BMI cutoffs. In a study of 1 513 Hong Kong Chinese, the risk (estimated by the odds ratio) of diabetes, hypertension, dyslipidemia, and albuminuria started to increase at a BMI of about 23 kg/m^2^, which is lower than the current WHO cutoff used to define an increase in morbidity among Europids [17], [22]. Furthermore, some obesity-associated complications may be particularly common in China. For example, although the prevalence of obesity in China (7.1%) [23] is relatively low compared with that in the United States (33.8%) [24], the prevalence of diabetes is similar across populations (9.7% for Chinese [25] versus 9.6% in whites [26]). This higher metabolic risk despite a lower BMI, concomitant with more total and trunk fat mass, provides evidence that can inform decisions regarding the merits of lower BMI cutoffs for defining overweight and obesity in China.

Recently, evidence has been accumulating that most complications of obesity develop not only on the basis of the degree of overweight but also according to the pattern of body fat distribution [27], [28]. Much data suggest that the central accumulation of body fat is more important than adipose tissue per se for the risk of developing complications [29]–[31]. Yet, central fat distribution was not considered in many previous multi-ethnic comparison studies [9], [21]. In the present study, higher metabolic risks were detected in Chinese people than in white people for a given BMI. Furthermore, this ethnic heterogeneity could be accounted for to a great extent by the ethnic difference in central fat distribution rather than total body fat. This finding is noteworthy, because the prevalence of abdominal obesity has increased in China between 1993 and 2009, from 8.5% to 27.8% among men and from 27.8% to 45.9% among women [32].

Several factors might be responsible for the ethnic disparities in fat and fat distribution. First, higher trunk-to-leg-length ratio was found in Chinese [33], and subjects with relatively longer trunk may have more trunk fat and body fat, as the mass per unit length of the trunk is higher than that of legs [34]. Second, Chinese tender to have smaller frame size [33]. Slender subjects have been reported to have less bone and connective tissue, and thus have higher percent body fat at given BMIs than Stockier subjects [35]. Third, physical activity may also provide a potential basis for this ethnic difference because variation in muscularity is a significant determinant of BMI in the population level even after adjusting for adiposity [27]. Finally, in addition to environmental factors, differences in genetic background between races may also contribute to the observed difference because body composition is strongly influence by genetic factors [29], [36]. However, despite all of these possible explanations of the results, the mechanisms of the ethnic difference in body composition remain unclear. Further study is needed to elucidate this disparity.

### Limitations

The results of this study should be interpreted in light of several limitations. First, the subjects, by necessity, were a convenience sample and may not be representative of the populations from which they were recruited. However, they were not specially selected, and the subjects from both sites covered a broad range of BMI and age. Second, the sample size for white adults in the present study was relatively small. However, a WHO expert consultation has indicated that meaningful body composition studies do not require large samples [37]. Third, only one man and one woman were used for the cross-validation of DXA measurements at Zhejiang University and Columbia University, and thus it is impossible to develop an equation to adjust for the potential DXA measurement bias. This limitation may in part be minimized by the fact that the both sites used the same type of DXA machine (Lunar Prodigy, Madison, WI, USA) and followed the same standardized measurement procedure. Fourth, no data on whites living in China or Chinese living in the United States was collected in the present study, so we were unable to investigate whether immigration affects the association of body fat with BMI and metabolic risk factors. Fifth, this is a post hoc analysis and the sampling time of the Chinese and white subjects were approximately 10 years apart. However, although the prevalence of obesity may change during this period, it is unlikely that this disparity in the time of data collection will dramatically affect the association of BMI with fat, fat distribution, and metabolic risk factors. Furthermore, due to the lack of data, lifestyles, such as physical activity and diet, were not adjusted in the present study. These factors may play an important role in body composition and should be taken into account in future studies. Finally, because DXA cannot distinguish visceral fat from subcutaneous fat, we could not examine the ethnic difference in intra-abdominal fat. Further studies using computed tomography or magnetic resonance imaging are encouraged.

### Conclusion

In summary, the present study demonstrated an ethnic difference in the association of BMI with fat (%BF) and fat distribution (%TF) and in the relationships of these variables to metabolic risk factors. Compared with white adults, Chinese adults had a greater proportion of fat, especially in the region of trunk, and it was this central fat distribution that could in part account for the higher metabolic complications in the Chinese. Effective strategies should be taken to reduce excess fat, especially abdominal fat, to prevent the chronic disease epidemic in China.

## Supporting Information

Figure S1 **Percentage body fat and percentage trunk fat by ethnicity in men and women.** Including subjects diagnosed with active disease (except for HIV) or on medication, excluding only those with missing DXA or blood test data. Error bars represent 95% confidence intervals. Data were analyzed by using analysis of covariance (ANCOVA) with “ethnicity” as the grouping variable and the listed variables as covariates. BMI, body mass index. *p<0.05, **p<0.01, ***p<0.001, for the difference with white men or women, respectively.
(TIF)

Figure S2 **Ethnic differences (Chinese - white) in metabolic risk factors in men.** Including subjects diagnosed with active disease (except for HIV) or on medication, excluding only those with missing DXA or blood test data. Bar heights indicate the mean difference, whereas error bars represent 95% confidence intervals. Data were analyzed by analysis of covariance (ANCOVA) with “ethnicity” as the grouping variable and the listed variables as covariates. Ht, height; BMI, body mass index; %BF, body fat divided by body weight; %TF, trunk fat divided by body fat.
(TIF)

Figure S3 **Ethnic differences (Chinese - white) in metabolic risk factors in women.** Including subjects diagnosed with active disease (except for HIV) or on medication, excluding only those with missing DXA or blood test data. Bar heights indicate the mean difference, whereas error bars represent 95% confidence intervals. Data were analyzed by analysis of covariance (ANCOVA) with “ethnicity” as the grouping variable and the listed variables as covariates. Ht, height; BMI, body mass index; %BF, body fat divided by body weight; %TF, trunk fat divided by body fat.
(TIF)
