# Supplementary figures and images for: Greater Abdominal Fat Accumulation Is Associated with Higher Metabolic Risk in Chinese than in White People: An Ethnicity Study
Source: PLoS One. 2013 Mar 14;8(3):e58688. doi: 10.1371/journal.pone.0058688 (PMC3597722; doi:10.1371/journal.pone.0058688)

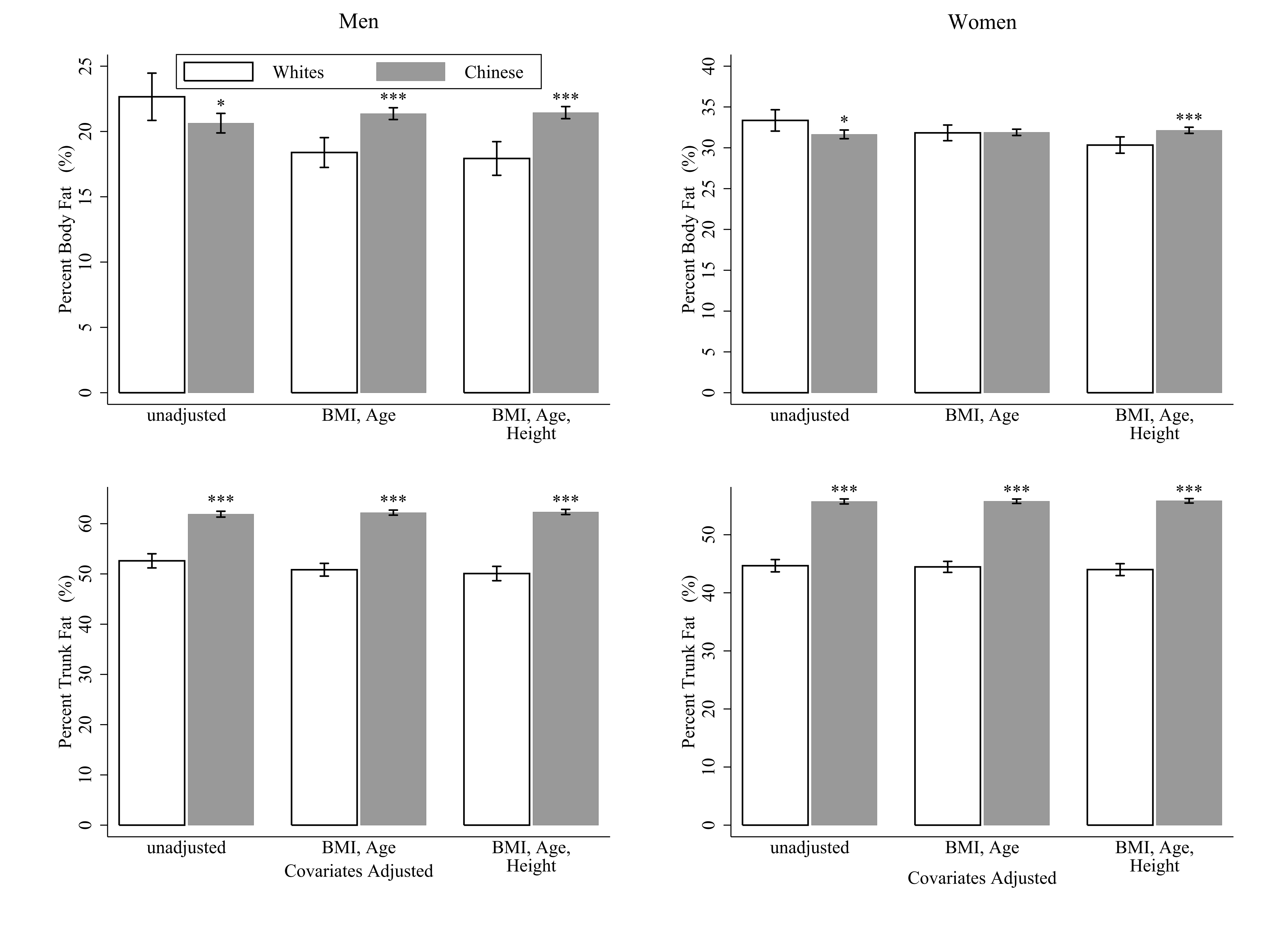

Supplement: Figure S1 — Percentage body fat and percentage trunk fat by ethnicity in men and women. Including subjects diagnosed with active disease (except for HIV) or on medication, excluding only those with missing DXA or blood test data. Error bars represent 95% confidence intervals. Data were analyzed by using analysis of covariance (ANCOVA) with “ethnicity” as the grouping variable and the listed variables as covariates. BMI, body mass index. *p<0.05, **p<0.01, ***p<0.001, for the difference with white men or women, respectively. (TIF) [file pone.0058688.s001.tif]

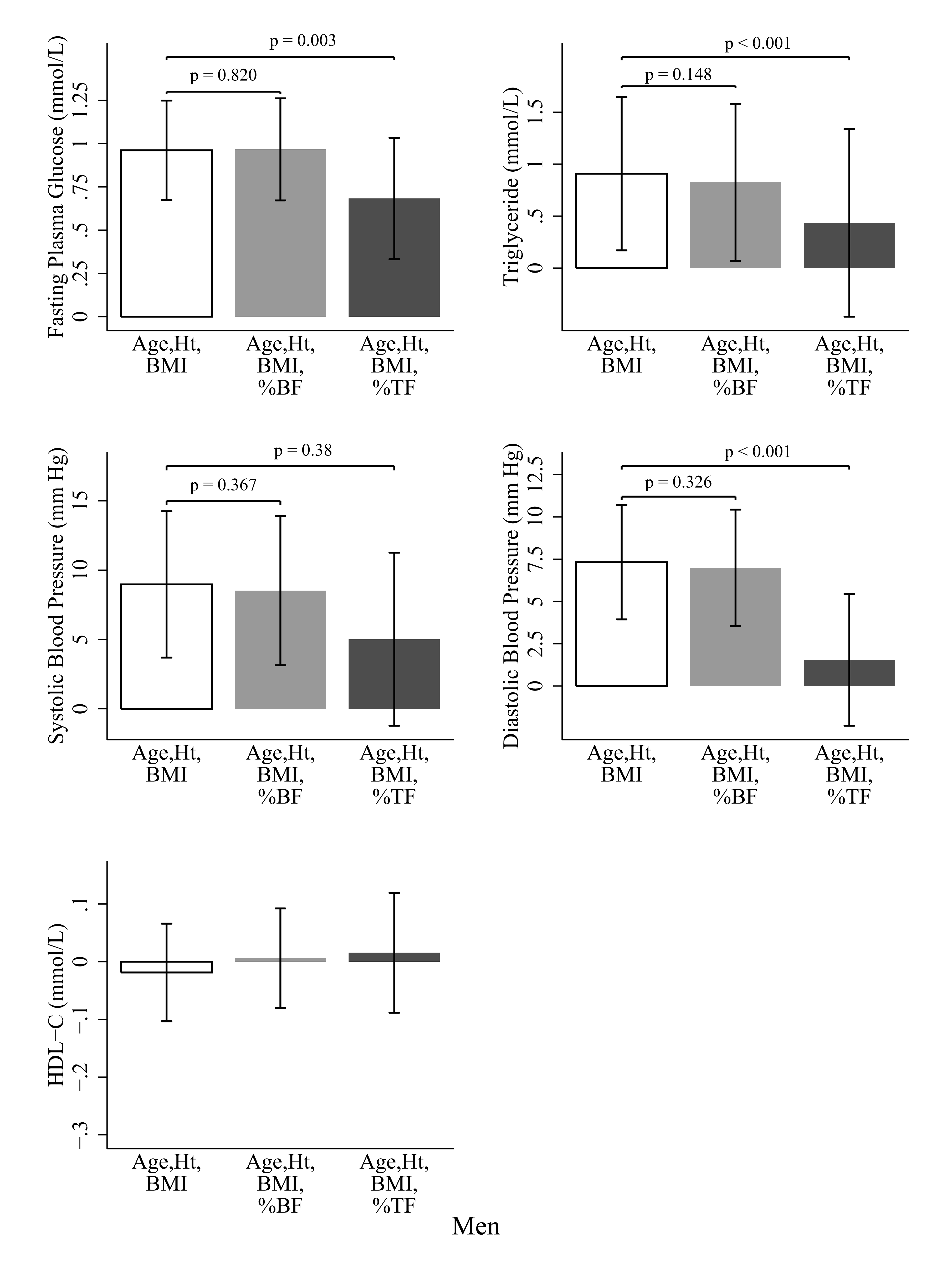

Supplement: Figure S2 — Ethnic differences (Chinese - white) in metabolic risk factors in men. Including subjects diagnosed with active disease (except for HIV) or on medication, excluding only those with missing DXA or blood test data. Bar heights indicate the mean difference, whereas error bars represent 95% confidence intervals. Data were analyzed by analysis of covariance (ANCOVA) with “ethnicity” as the grouping variable and the listed variables as covariates. Ht, height; BMI, body mass index; %BF, body fat divided by body weight; %TF, trunk fat divided by body fat. (TIF) [file pone.0058688.s002.tif]

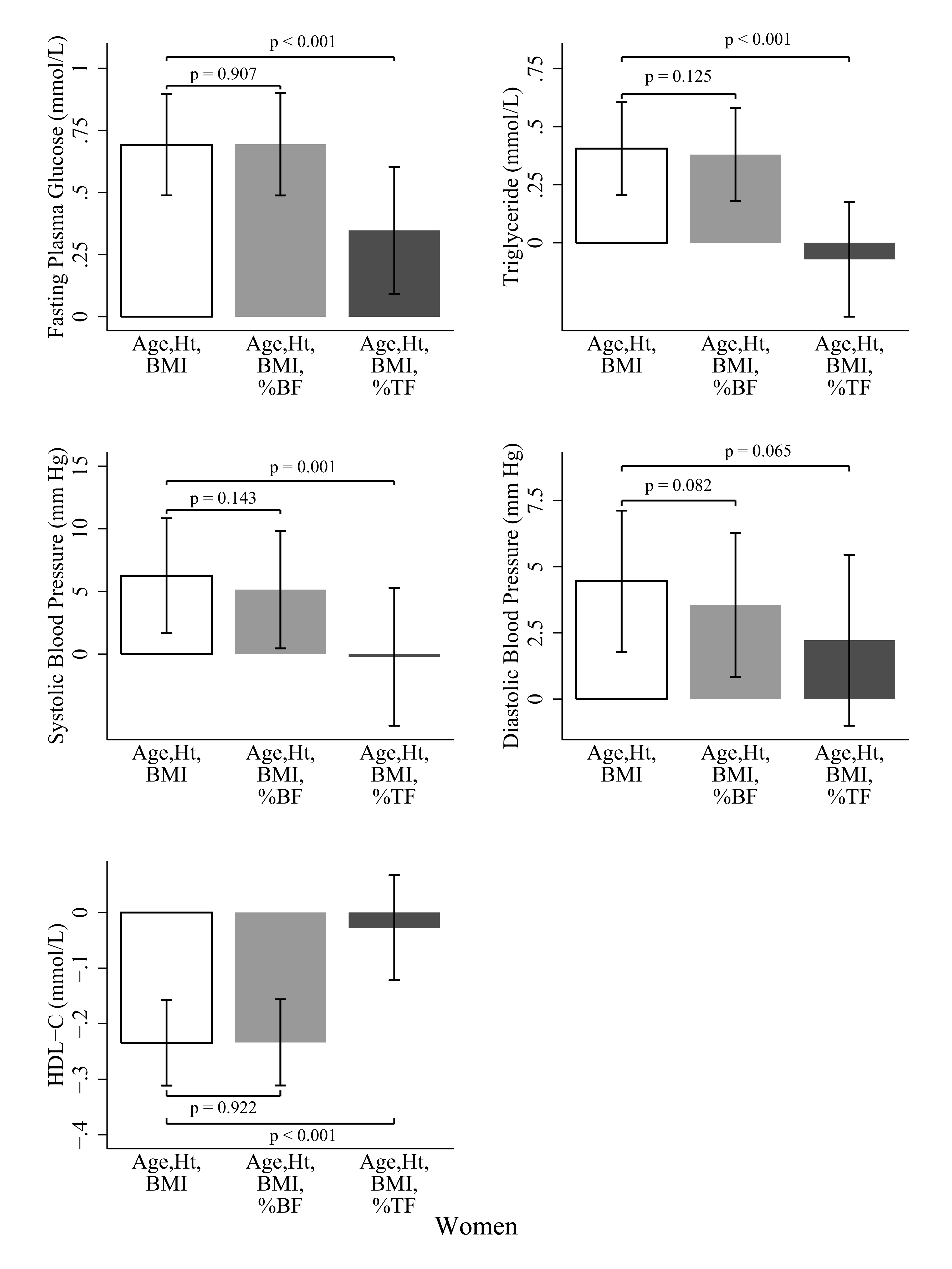

Supplement: Figure S3 — Ethnic differences (Chinese - white) in metabolic risk factors in women. Including subjects diagnosed with active disease (except for HIV) or on medication, excluding only those with missing DXA or blood test data. Bar heights indicate the mean difference, whereas error bars represent 95% confidence intervals. Data were analyzed by analysis of covariance (ANCOVA) with “ethnicity” as the grouping variable and the listed variables as covariates. Ht, height; BMI, body mass index; %BF, body fat divided by body weight; %TF, trunk fat divided by body fat. (TIF) [file pone.0058688.s003.tif]
